# Learning on the Fly: The Interplay between Caspases and Cancer

**DOI:** 10.1155/2018/5473180

**Published:** 2018-04-29

**Authors:** Derek Cui Xu, Lewis Arthurton, Luis Alberto Baena-Lopez

**Affiliations:** ^1^Sir William Dunn School of Pathology, University of Oxford, Oxford OX13RE, UK; ^2^Cell Biology Section, National Institute of Dental and Craniofacial Research (NIDCR), National Institutes of Health (NIH), Bethesda, MD, USA

## Abstract

The ease of genetic manipulation, as well as the evolutionary conservation of gene function, has placed* Drosophila melanogaster* as one of the leading model organisms used to understand the implication of many proteins with disease development, including caspases and their relation to cancer. The family of proteases referred to as caspases have been studied over the years as the major regulators of apoptosis: the most common cellular mechanism involved in eliminating unwanted or defective cells, such as cancerous cells. Indeed, the evasion of the apoptotic programme resulting from caspase downregulation is considered one of the hallmarks of cancer. Recent investigations have also shown an instrumental role for caspases in non-lethal biological processes, such as cell proliferation, cell differentiation, intercellular communication, and cell migration. Importantly, malfunction of these essential biological tasks can deeply impact the initiation and progression of cancer. Here, we provide an extensive review of the literature surrounding caspase biology and its interplay with many aspects of cancer, emphasising some of the key findings obtained from* Drosophila* studies. We also briefly describe the therapeutic potential of caspase modulation in relation to cancer, highlighting shortcomings and hopeful promises.

## 1. Introduction

As the second leading cause of death worldwide, cancer claimed the lives of nearly 9 million individuals in 2015 (http://www.who.int). Consequently, a great deal of effort has been expended towards understanding all aspects of tumorigenesis and potential treatments. As part of these efforts, recent investigations have linked some of the defining traits in carcinogenesis, or “hallmarks of cancer,” with the deregulated activity of cysteine-aspartic proteases known as caspases [1–11]. In particular, it has been shown that caspase malfunctions could be crucial for explaining tumour cells' ability to evade cell death mechanisms [6, 7], to promote tumour-enabling inflammation and avoid immune destruction [3, 4, 11], to maintain high rates of cell proliferation without entering into the cell differentiation program [2, 10, 12, 13], and to metastasize [5, 14, 15]. However, the molecular basis linking the activity of caspases with these tumorigenic properties is not fully understood. Here, we review studies connecting the activity of these enzymes with different aspects of carcinogenesis, dedicating special attention to some of the key findings obtained from different* Drosophila* models.

**Figure 1:**
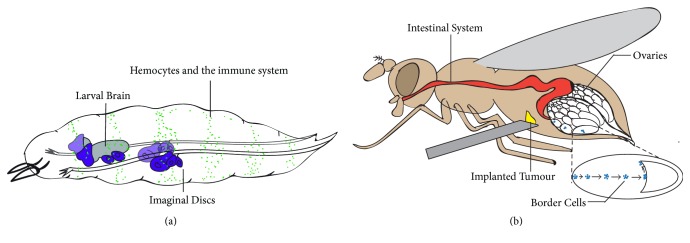
Schematic diagram showing a* Drosophila* larva (a) and an adult fly (b). (a) The larval brain (grey in (a)) and the imaginal discs (purple in (a)) have often been genetically manipulated to induce metastatic tumours with physiological relevance in humans. The immune system (green dots in (a)) represent the macrophage-like* Drosophila* cells, hemocytes. Hemocytes have been used to study immune responses and tumour associated inflammation (a). (b) Recent studies have exploited systems in the adult fly to investigate metastatic and tumorigenic properties. Adult ovaries (white in (b)) are often used for testing the invasive ability of implanted tumours (originating from imaginal discs or the larval brain) in the abdomen (yellow in (b)). The natural migratory ability of ovarian border cells (blue in (b)) has been used to decipher the molecular mechanisms of cell migration during development. The* Drosophila* intestinal system (red in (b)) is a well-established system for modelling many aspects of tumorigenesis related to colon carcinomas.

For over a century, the fruit fly has proven to be an effective model organism to study a wide range of biological phenomena and carcinogenesis (Figure 1) [16, 17]. Beyond the practical advantages for maintaining this insect in laboratory conditions (e.g., low cost, short life cycle, and high breeding rate), several other reasons posit this model organism at the forefront of genetic research.* Drosophila* contain a simpler and less redundant genome compared to humans, while preserving 77% of genes relevant for human disease [18, 19]. They also possess an extremely versatile set of genetic tools for manipulating gene expression with spatiotemporal control (Gal80/Gal4/UAS, QS/QF/QUAST, and Gal80/LexA/LexOP systems), accurate systems for generating genetic mosaics (FLP/FRT, CRE/LoxP systems), readily available methods for incorporating stable genetic elements into the genome (P-element random transformation, specific integration using attP/attB recombination sites), and genome editing techniques with base-pair precision (CRISP/Cas9 and homologous recombination) [20, 21]. These advantages have enabled the identification of many oncogenes, tumour suppressors, and signalling components using* Drosophila* cellular models [17]. Similarly, fly research has provided key insights about caspase biology.

Caspases were first discovered in* Caenorhabditis elegans *as regulators of cell death and, later, were implicated in the regulation of inflammation [22–24]. Caspase-mediated apoptosis is an essential process in multicellular organisms that helps to control organ size, shape, and tissue homeostasis, through the elimination of unnecessary or unhealthy cells [25]. All members of this protein family are synthesized as inactive zymogens (procaspases), and only after several steps of proteolytic processing do they become fully active [26]. Structurally, caspases contain two subunits that form the catalytically active pocket. In addition, some members contain N-terminal protein recruitment domains (DEDs or CARDs), which facilitate the formation of large protein complexes (e.g., apoptosome, inflammasome, and PIDDosome) essential for their efficient activation [26, 27]. Caspases can be subdivided into two categories depending on their temporal activation during the process of apoptosis. Initiator/apical caspases are activated at early stages of apoptosis and, immediately after, trigger the enzymatic activation of effector/executioner members [9]. During apoptosis, high levels of caspase activation can enzymatically cleave a plethora of protein substrates throughout all subcellular compartments, thus leading to the stereotyped disassembly of organelles and subsequent shutdown of all essential cellular tasks [27]. In* Drosophila *the apical caspases are encoded by the genes* death regulator Nedd2-like caspase (dronc)*,* death related ced-3/Nedd2-like caspase (dredd)*,* and Ser/Thr-rich caspase (strica)*, while the executioner members are* death related ICE-like caspase (drice)*,* death-caspase-1 (dcp-1)*,* death executioner caspase related to Apopain/Yama (decay)*, and* death associated molecule related to Mch2 caspase (damm)* [28]. As suggested by their nomenclature, caspases are tightly regulated to prevent the inadvertent activation of apoptosis. This regulation does not rely exclusively on enzymatic processing, but often demands different post-translational modifications (e.g., phosphorylation, ubiquitination) [29, 30], as well as transient interactions with regulatory protein partners: inhibitors of apoptosis proteins (IAPs), or pro-apoptotic factors* head involution defective (hid), reaper (rpr), grim (grim)*, and* sickle (skl)* [28]. Beyond their apoptotic role, caspases have recently been implicated in a broad range of non-lethal activities, including the regulation of the immune response [3, 4, 7, 31], stem cell properties [10, 12], cell differentiation [13], cell migration [5, 32], and intercellular communication [12, 14, 33–35], though little is known about these novel non-apoptotic functions. Therefore, if deregulated, caspase activity can contribute to almost every step of tumorigenesis (overproliferation, evasion of cell death and immune destruction, tumour-promoting inflammation, and metastatic invasion). This manuscript aims to provide key examples of what we have learned from* Drosophila *models about the interplay between caspases and cancer.

## 2. Caspase-Aided Survival and Proliferation of Tumoural Cells

Fundamental to the pathological progression of cancer is the capacity of tumorigenic cells to excessively proliferate while escaping apoptotic death [1]. Therefore, it is not surprising that insufficient caspase activation is one of the defining features of cancerous cells [6–8, 36–38]. Indeed, the evasion of cell death has been identified as a major risk factor during tumorigenesis, providing faulty cells the autonomy to undertake uncontrolled proliferation [36–38]. However, the recent descriptions of non-lethal functions associated with caspases [10–12, 14, 33, 35, 39–41] suggest a more complex intersection between these enzymes and tumorigenesis. Some of the newly identified caspase functions alter the tumorigenic cells' ability to grow and differentiate, while others can influence the cellular microenvironment non-cell autonomously, thus facilitating the cellular selection and proliferation of transformed cells. This section of the manuscript describes selected examples regarding key phenomena regulated by caspases that directly or indirectly enable the clonal expansion of tumorigenic cells.

### 2.1. Evasion of Cell Death

**Figure 2:**
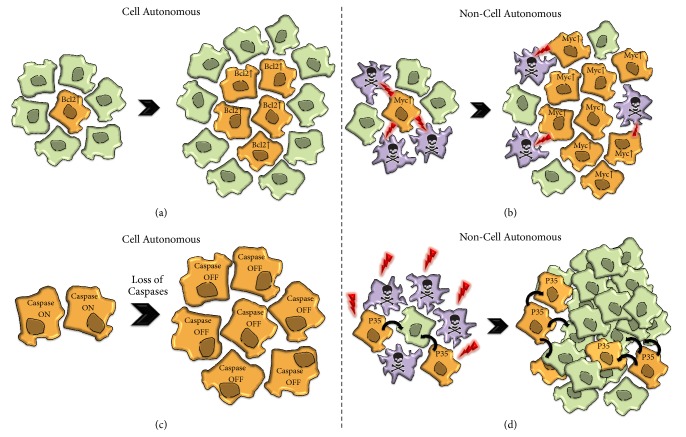
Examples of cellular phenomena that contribute to the clonal expansion of tumour cells. (a) Cancerous cells evade apoptosis through the upregulation of prosurvival proteins such as BCL-2, thus facilitating their clonal expansion. (b) The complex phenomenon of cell competition enables the elimination of slow-proliferating cells (purple), if confronted with fast-proliferating Myc-expressing cells (orange). Lightning symbols indicate the lethal effect (skull symbol) of Myc-expressing cells (orange) on surrounding neighbours (b). (c) Caspase activation defects in the* Drosophila *proneural clusters promote an excess of sensory organ precursor cells. The non-apoptotic activation of the caspase cascade via Drice leads to cleaved Shaggy, thus modulating the number of sensory organ precursors (c). (d) Drawing showing a non-cell autonomous caspase-mediated phenomenon that facilitates tumorigenesis. Following ablation of cells though irradiation (red lightning symbol) most of cells die (d). If apoptosis is impeded in such a scenario, by ectopic expression of P35, the so-called undead cells (in orange) release pro-proliferative signals (black arrows) into surrounding neighbours (in green), thus instigating tumour formation (d). The dashed line separates examples in which caspases have cell autonomous versus non-cell autonomous effects.

As popularized by the “hallmarks of cancer” paradigm, a fundamental aspect of cancer initiation and progression is the avoidance of cell death [1]. The literature encompassing this topic is extensive and far beyond the scope of this review. However, it is clear that transformed cells are often resistant to apoptosis due to defects in caspase activation, mainly from the upregulation of prosurvival genes or downregulation of pro-apoptotic factors [6, 7, 42–47]. Members of the anti-apoptotic family of BCL-2 such as Mc1-1 and BCL-XL are commonly overexpressed in cancer, thus resulting in enhanced tumour progression and poor patient prognosis (Figure 2(a)) [6, 7, 42, 43]. Conversely, downregulation of pro-apoptotic proteins such as BAX is often inactivated in colon carcinomas and specific subtypes of breast cancer [44–47]. Different examples obtained from* Drosophila* studies have not only confirmed these theories, but also provided key molecular details towards our understanding of how some types of tumours prevent the apoptotic programme.

The tumour-suppressor signalling cascade referred to as Hippo pathway was delineated in* Drosophila *[48]. However, some years before its formal description, a link had already been described between one of the key members of the pathway* (Mst-1)* and caspases. In particular, it was observed that the caspase-3-mediated cleavage of Mst-1 had pro-apoptotic effects [49] and facilitated chromatin condensation [50]. On the other hand, it was described that the same biochemical events had a pro-differentiating role in skeletal muscle progenitor cells [51]. In* Drosophila*, the activation of the Hippo pathway normally prevents the translocation of the transcriptional activator Yorkie (Yki) into the nucleus and the subsequent activation of target genes. Whereas some of the Yki target genes promote cell division (e.g., Cyclin-E and Myc) [48, 52–57], others are potent inhibitors of apoptosis (e.g., the* Drosophila* inhibitor of Apoptosis 1 (Diap-1) and the* bantam* microRNA) [55, 58, 59]. The regulatory regions of the* diap-1* locus contain binding sites for the Yorkie-Scalloped (Yki-Sd) complexes, which potently stimulate the transcription of the gene upon binding [52, 55–57]. In turn,* bantam* can post-transcriptionally bind to the mRNA of the pro-apoptotic factor Hid, triggering its degradation [60]. Furthermore, the Hippo complex can also limit the activity of the caspase-2/9 ortholog in flies, Dronc [61]. These effects collectively facilitate the survival and rapid clonal expansion of Yki-activating cells. A further example illustrating the mechanisms of cell death evasion present in tumour cells was obtained investigating the ectopic activation of the Epidermal Growth Factor (EGF) signalling pathway. EGF signalling deregulation often correlates with tumour overgrowth and metastasis [62, 63]. Different studies have shown that, upon EGF activation, pro-apoptotic genes such as Hid are transcriptionally repressed [64]. Furthermore, post-translational inhibitory phosphorylation events also prevent the function of Hid [65]. As previously described, these effects promote cell survival and, ultimately, proliferation of EGF-activating cells. Importantly, most of the signalling pathways deregulated in tumours often crosstalk between themselves in a context-dependent manner (e.g., EGFR signalling regulates the Hippo pathway in mammals by phosphorylating the Yki-like protein YAP) [66]. In tumorigenic situations, this complicates the interpretation of their biological effects, in terms of survival and proliferation.

### 2.2. Caspases as Key Regulators of Cell Competition

The phenomenon of cell competition was first described in* Drosophila *around 40 years ago through the detailed analysis of wild-type genetic mosaics in heterozygous flies for the* Minute *genes [67–70]. The* Minute *genes encode for several ribosomal proteins that impede protein biosynthesis in mutant conditions. Although* Minute* heterozygous flies are phenotypically normal [68], heterozygous cells proliferate at a slow rate and are selectively eliminated if surrounded by wild-type cells [68, 70]. Importantly, without changing the final size of organs, this process facilitates the clonal expansion of faster-proliferating cells (winner cells) and the simultaneous elimination of slower-proliferating cells (loser cells) via apoptosis [70, 71]. Loser cells can be readily identified at the final stages of the elimination process by the activation of cell death markers such as cleaved caspase-3 and the apoptosis assay TUNEL [72]. Furthermore, recent work by Levayer and coauthors also indicates that caspase activation could precede the delamination of loser cells from tissues [73]. Notably, the suppression of caspase activation can strongly suppress the phenomenon and ultimately the tissue colonization of faster-dividing cells [74]. Considering the scope of this review, a key finding was the discovery that the upregulation of the growth factor Myc (commonly found to be deregulated in cancers) [75] and other tumorigenic-promoting conditions (e.g., combined upregulation of EGFR pathway and loss of cell polarity, or the Hippo pathway) are able to exploit this phenomenon for unrestrained clonal expansion (Figure 2(b)) [76–78]. In recent years, a vast amount of literature has emerged demonstrating the evolutionary conservation of the phenomenon from worms to mammals and some of the molecular pathways implicated in the process [70, 79, 80]. Cell competition has thus been hypothesised to partake in the selection of cancerous cells in tumorigenesis [70, 81–88]. Reciprocally, it has been suggested that cell competition could act as a tumour suppressing mechanism when wild-type cells have the ability to outcompete potentially dangerous cells [70, 89]. Since a fundamental component of cell competition is caspase activation in loser cells, it is conceivable that tumours can take advantage of this biological phenomenon to grow, through blocking caspase activation autonomously, or abnormally triggering it in the wild-type surrounding neighbours.

### 2.3. Cell Autonomous Caspase-Mediated Regulation of Cell Proliferation

Beyond affecting cell death, caspase deregulation could compromise the activity of key signalling pathways (e.g., Hippo, Notch, TGF-*β*, and JAK-STAT) and cell cycle regulators (e.g., p21, p27, and cyclin-D2) promoting tumour cell proliferation in many organisms [90–93]. In* Drosophila*, caspase-3-like activation (mediated by the Hippo pathway) has been demonstrated to cleave the chromatin remodelling protein, Brahma, reducing intestinal cell proliferation [94]. Therefore, in this cellular context, caspase defects are associated with the clonal expansion of intestinal precursor cells upon damage [94, 95]. Moreover, in the* Drosophila* brain, protein-protein interactions between Dronc and the Notch signalling regulator, Numb, block the activity of the latter, preventing unrestrained cell proliferation [96]. The caspase-mediated regulation of cell proliferation appears to be conserved from* Drosophila *to mammals. Kennedy and collaborators demonstrated a decrease in the proliferation of human T-cells following application of caspase inhibitors [97]. These defects were also correlated with flaws in the regulation of the cell cycle proteins p21, p27, and cyclin-D2 [93, 98]. Paradoxically, current literature also suggests that caspases could limit proliferation in tumorigenic scenarios, inducing the expression of cell proliferation inhibitors; caspase-7 reduces proliferation in breast cancers through the downregulation of the cell cycle regulator p21^cip^ [92]. The explanation for these opposing roles, and how this discrepancy occurs, is still unknown.

### 2.4. Regulation of Caspase-Dependent Stem Cell Function and Differentiation

The proliferative potential of cells can also be maintained though the regulation of cell differentiation. Indeed, the act of differentiation itself could be considered a powerful mechanism for limiting tumour growth. Importantly, caspases are emerging as potent controllers of stem cell properties, as well as differentiation factors [12, 13]. In the* Drosophila *proneural clusters, the sequential activation of the different members of the caspase cascade (Dark > Dronc > Drice) leads to a cleaved form of the fly homolog of GSK-3,* shaggy46 (sgg)*. This caspase-dependent event limits the number of sensory organ progenitor cells without affecting their cell viability [99]. Accordingly, loss-of-function mutations in either the aforementioned caspases or* sgg* generate an excess of sensory organ precursors and neurogenic defects (Figure 2(c)) [100]. Further highlighting the relationship between differentiation and the apoptotic program, it has been reported that the expression of the transcription factor Cut simultaneously promotes differentiation and inhibits apoptosis [101]. The authors suggested that this regulation prevents the expansion of cancer cells through the removal of uncommitted precursors* in statu nascendi* [101]. Interestingly, the cell death regulatory role of Cut is conserved in vertebrates, and Cux1 human cancer cells show apoptotic defects. Many examples have been identified supporting the implication of caspases in the regulation of embryonic and adult stem cell properties [12, 13, 51, 99, 102, 103]. Conversely, it has also been shown that caspases can revert the differentiation state of specific cell types to generate induced-pluripotent stem cells (e.g., generation of induced-pluripotent stem cells from differentiated fibroblast [104]).

Taking into account all the evidence, it is conceivable that caspase deregulation may partake in the aberrant differentiation of cancerous cells. Indeed, direct examples of such exist. Downregulation of caspase-9 results in poorly differentiated colon malignancies, whereas its upregulation results in highly differentiated tumours with decreased proliferation and increased apoptosis [105]. Furthermore, expression of cleaved caspase-3 is a common feature of advanced cancer stages associated with aberrant differentiation of the cancerous cells [106]. More controversial is the role of caspase-14 in cancer pathology [107, 108]. Despite the tentative correlation between caspase-regulated differentiation and cancer pathologies, the biochemical interactors orchestrating these tumorigenic phenotypes are largely unknown.

### 2.5. Remote Caspase Effects Facilitating Tumorigenesis

In addition to the cell autonomous caspase-regulated effects, these enzymes can also contribute to tumoural transformation through non-cell autonomous mechanisms. Recent investigations have uncovered the phenomenon of apoptosis-induced cell proliferation (AiP) [109]. This phenomenon encompasses all forms of induced proliferation facilitated by the activation of caspases and is crucial for ensuring homeostatic cell numbers within organs and the regenerative process [10, 11, 74, 109–113]. Seminal studies in* Drosophila *demonstrated that high doses of ionizing irradiation during larval stages could eliminate more than 50% of the prospective imaginal epithelial cells; however, healthy full-size adult flies emerged [111, 114]. Interestingly, the artificial suppression of effector caspase activity upon triggering the caspase pathway (e.g., irradiation) generates large hyperplastic phenotypes (Figure 2(d)) [10, 115, 116]. Importantly, the hyperplasia and the regeneration process are severely compromised upon blocking the upstream component of the caspase cascade, Dronc [115, 117, 118]. These observations suggested that caspase-activating cells were releasing mitogenic signals in order to promote tissue regeneration, which can lead to tumour formation if these cells are not effectively eliminated [10, 11, 74, 110, 111, 113, 116]. Although the biological nature of these mitogenic signals is not fully understood and likely context dependent, it is becoming apparent that pro-inflammatory molecules and the production of reactive oxygen species could participate in this process (see Section 4). It is unknown whether caspase-9 in mammals shares a comparable ability to induce apoptosis-induced proliferation like its fly counterpart, Dronc [116]. However, caspase-3 is commonly downregulated in particular cancers [119]; if correlated with the activation of upstream caspase components, this may lead to the promotion of abnormal growth in the wild-type surrounding cells.

## 3. Caspase-Aided Cell Migration and Metastasis

**Figure 3:**
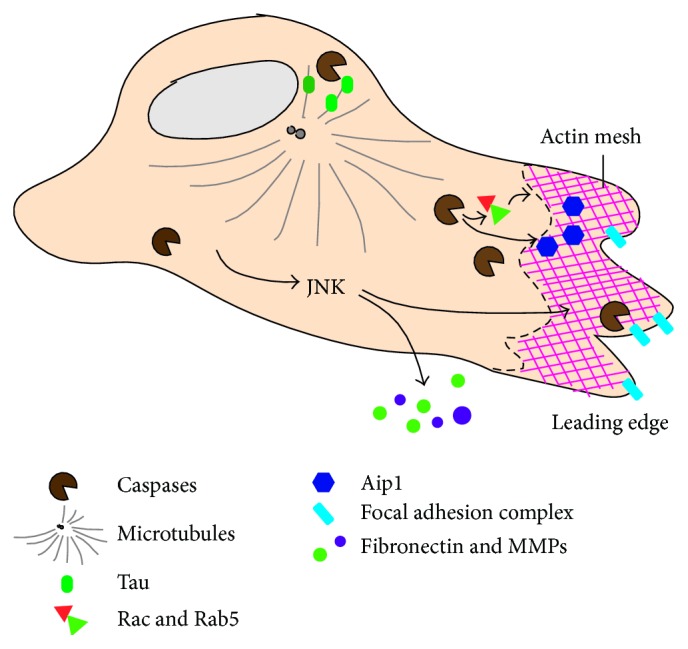
Graphic summary of caspase effects in different subcellular locations during cell migration and metastasis. Caspases are known to regulate the cytoskeleton remodelling elements crucial for migration (such as the microtubule-stabilizing protein tau and actin regulators Aip1, Rab5, and Rac), as well as modulating the stability of focal adhesion complexes. They also modulate the secretion of factors into the ECM that facilitate invasion and migration (MMPs and fibronectin).

While overproliferation and the evasion of cell death are some of the most fundamental traits of cancer cells [1], the spreading of transformed cells from the primary tumour to other sites of the body (metastasis) is one of cancer's most deadly attributes. Indeed, the vast majority of deaths related to cancer result from the appearance of secondary tumours called metastases [120]. Because of this, a great deal of effort has been expended towards understanding the invasion mechanisms and the metastatic process. The invasion and colonization in metastasis require the detachment of cells from neighbours through the disruption of cell-cell contacts, degradation of the surrounding extracellular matrix (ECM), and extensive remodelling of the cytoskeleton [121, 122]. Under normal conditions, these cellular tasks are tightly regulated; however, in cancer cells such regulation is commonly perturbed [123–129]. This section of the manuscript compiles some of the key findings relating the activity of caspases with cell migration and metastasis of transformed cells (Figure 3).

### 3.1. Drosophila Models Linking Caspases, Migration, and Metastasis

During apoptosis, dying cells undergo major cytoskeletal reorganization that demands caspase-mediated pathways (Figure 3) [130]. Additionally, caspases are known to directly modify intercellular attachments by modulating the turnover of cell adhesion molecules (Figure 3) [129, 131, 132]. They can also indirectly affect the secretion of inflammatory factors and matrix metalloproteinases (MMPs) to degrade the ECM (Figure 3) [133–135]. Collectively, this supports the hypothesis that caspases play a key role in regulating the cellular motility in normal and metastatic cells [32, 135]. Support for this hypothesis has been obtained from different organisms, including flies.

A* Drosophila* model describes how the simultaneous activation of caspases and the inhibition of cell death through the effector caspase inhibitor P35 facilitate cell extrusion and spreading of wing imaginal cells [134]. This work attributes the invasive ability of the genetically modified cells to the non-apoptotic activity of Dronc and the downstream activation of the c-Jun N-terminal kinase (JNK) signalling pathway. Importantly, these factors induce the expression of the matrix metalloproteinase-1 (MMP-1), which ultimately degrades the ECM and basement membrane [134]. MMP production is also observed in* Drosophila *transplantation models of metastasis, in which larval metastatic brain tumours are transferred into the abdomen of host adult flies. Once transplanted, tumours in the abdominal cavity of the host can metastasize into other tissues, such as the ovary [136, 137]. This is a particularly powerful assay that can be used to highlight the differences in metastatic potential arising from different tumour-inducing mutations [137].

Another* Drosophila* model conventionally used to study cell migration and metastasis relies on the ovaries and a collection of follicle cells within the egg chamber, called the border cells, which show invasive and migratory properties [138]. Border cells rearrange their cytoskeleton, cell polarity, and adhesive properties to detach from the epithelium and migrate towards the namesake border of the developing oocyte [138]. Many of the pathways governing this migratory process share strong similarities with the metastatic behaviour of many human cancer cells [138–140]. Importantly, this model also began to shed light on the role of caspases during metastasis, when it was reported that the overexpression of Diap-1 rescued the migration defects caused by a dominant negative mutant for the GTPase Rac [139]. Evidence also indicated that Diap-1 could directly interact with Rac and profilin to regulate actin dynamics. Simultaneously, it was reported that low levels of Dronc activation could have an inhibitory effect on the migration of border cells [139].

### 3.2. Caspase Implication during Physiological Cell Migration and Metastasis in Mammalian Models

As in* Drosophila*, there is solid evidence suggesting the prominent role of caspases in physiological cell migration and the metastatic behaviour of mammalian cells. In physiological conditions, caspase-11 has been shown to interact with the actin-interacting-protein-1 (Aip1) to promote actin depolymerisation and cell migration [141]. Correlated with this observation, caspase-11-deficient macrophages show reduced motility [141]. Caspase-8 is also heavily implicated in cell migration and metastasis [142], and Caspase-8 knockout mouse embryonic fibroblasts (MEFs) are unable to form actin-based lamellipodia, leading to defective integrin-mediated cell motility [143]. Additionally, caspase-8 has been observed to be recruited and localized to leading lamellae in endothelial cells [144], as well as the leading edge of actin-based lamellae at focal adhesion complexes in neuroblastoma cells [145]. Interestingly, this promotes cell migration through a mechanism independent of its protease activity on effector caspases [144, 145]. This is not surprising, since many of the caspase-8 pro-migratory effects could be mediated by the modulation of actin-dynamics regulators such as Rac and Rab5 [143, 146, 147]. However, the lack of caspase-8 may also promote migratory behaviour. Loss of caspase-8 activity is known to have a major role in activating anoikis, a form of programmed cell death activated by the detachment of epithelial cells from the ECM, in a variety of cancer types [148, 149]. Since the development of anoikis resistance is critical for tumour metastasis [150, 151] and loss of caspase-8 in cancers compromises the apoptosis triggered during anoikis [151], it could be interpreted that caspase-8 pro-migratory effects during metastasis are an indirect consequence of aiding cell survival. Supporting this hypothesis, it has been shown that caspase-8 deficiency also promotes the dissemination of implanted cancerous cells in the embryonic chick due to a lack of cell death [152]. However, caspase-8 deficiency in a mouse neuroblastoma model led to a significant increase in metastases, due to ECM structural changes and production of inflammatory cytokines such as TGF-*β* [153]. These findings collectively indicate a complex and context-dependent intersection between caspase-8 and cell migration/metastasis.

Caspase-3 has also been linked to the process of cell migration in physiological and metastatic scenarios. It has been shown that the neuronal microtubule-stabilizing protein Tau is cleaved by caspase-3 in PC12 cells [154]. Caspase-mediated cleavage of Tau then enables the dispersion of these cells, suggesting that caspase-3 activity may regulate the cytoskeleton disassembly required for neuronal precursors to migrate towards their destinations [154]. Procaspase-3 was also found to have an inhibitory role in fibronectin secretion, and MEFs deficient for caspase-3 show increased adhesion to substrates and decreased migration velocity in wound-healing assays [155]. Interestingly, these regulatory capabilities were independent of caspase-3's catalytic activity, as the decreased migration velocity and increased adhesion of caspase-3 deficient MEFs were rescued following introduction of a catalytically dead version of the protein [155]. These results suggest a promigratory role for caspase-3 independent of its enzymatic action. In metastatic scenarios, caspase-3 has been shown to play a pro-migratory role. Whereas caspase-3 inhibition reduces glioblastoma motility and invasiveness [156], its activation promotes migration and invasion in ovarian, melanoma, and hepatoma cancer cells [157–159]. However, conflicting evidence also suggests that caspase-3 could be an inhibitory factor in stroke-induced migration and neurogenesis [160]. Altogether, the described findings illustrate that caspase roles in cell migration and metastasis are far from straightforward and highly context dependent.

## 4. Caspase-Aided Evasion of Immune Destruction and Tumour-Promoting Inflammation

Components of both the innate and adaptive immune system have been located in virtually every type of tumour [161], often making the tumour's environment mirror that of a physiological inflammatory response [162]. Initially, it was thought that the presence of immune cells indicated the body's attempts to eliminate the tumour; however, it is now apparent that the immune response and resulting inflammation can have a stimulating effect on tumour growth and cancer progression [1]. Exactly how cancers evade immune destruction and instead highjack specific immune responses to promote their own growth is an intense subject of research. However, it is clear from decades of work that the release of bioactive molecules from immune cells can contribute towards every step of tumorigenesis (e.g., enhanced growth, angiogenesis, and initiation of metastatic programs) [163–165]. Indeed, tumour-promoting inflammation is now considered a core enabling characteristic of cancer, and the evasion of immune destruction has joined the ranks of other cancer hallmarks [1].

Since the original association of caspase-1 with the inflammation process in mammals [166, 167], intense research efforts have been devoted to understanding the role of the so-called “inflammatory” caspases in macrophages and other immune cells [168–170]. The primary function of this subgroup of caspases appears to be regulating the maturation and release of proinflammatory cytokines responsible for the inflammatory response [3, 168, 171]. Additionally, inflammatory caspases are potentially involved in the dampening and sequestering of proinflammatory signals released by infected and tumorigenic cells [4, 172]. Despite the fact that classical inflammatory caspases have not been described outside of vertebrates [170],* Drosophila* is known to be a useful model for investigating the immune response. Signalling and transduction pathways are conserved, and analogous elements of the immune system exist [173]. While the presence of a primitive form of adaptive immunity is still under debate [174–176], the* Drosophila* innate immune system shares many similarities with ours and conserves most of the elements and signalling pathways implicated in the cellular and humoural responses (e.g., Toll/IL-R, NF-kB, and Eiger/TNF-*α*) [173, 177, 178]. The innate* Drosophila* immune system is also sufficient for providing immune surveillance, while producing the proinflammatory responses associated with wound healing, pathogen defence, and tumour response [179–181]. Hemocytes are the circulating immune cells in* Drosophila* analogous to the phagocytic mammalian macrophages [173]. Like their mammalian counterparts, hemocytes are responsible for a large cohort of cellular immune responses, including the clearance of apoptotic bodies in tissue damaged areas, production of signalling molecules, and encapsulation/elimination of pathogens, and are recruited to tumours [180]. Additionally, the* Drosophila* immune response is largely caspase-dependent [182–187]. In response to specific pathogens and tissue damage, the* Drosophila* caspase* dredd* is essential for triggering immune responses through the activation of the key transcriptional factor NF-kB [183–185, 188, 189]. Despite not being formally included in the group of inflammatory caspases, the mammalian homolog of* dredd*, caspase-8, has strong links to inflammatory processes through RIPK activity in normal cells and in transformed cells through the release of inflammatory exosomes [3, 190]. Additionally, the “apoptotic” caspase Dronc has also been associated with the inflammatory response [182]. These similarities between flies and mammals make a strong case for considering* Drosophila* as a viable model for investigating the interplay between caspases, the immune response, and cancer.

**Figure 4:**
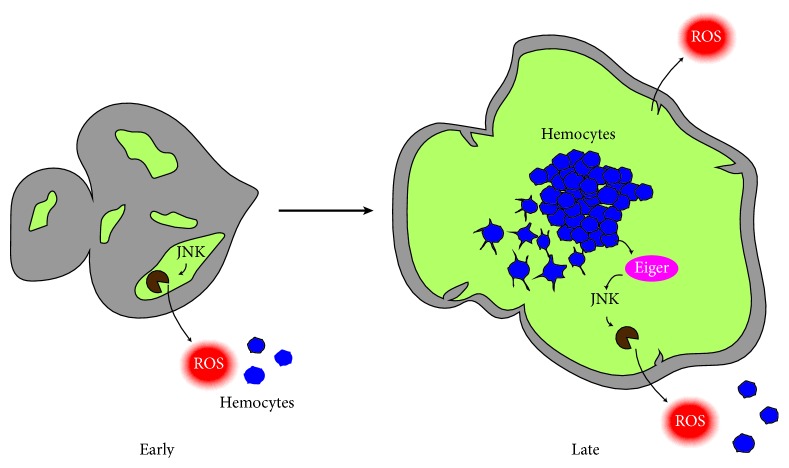
Schematic diagram showing a recently developed model of clonally induced tumours in* Drosophila* eye imaginal discs. During early stages of tumorigenesis, cancerous cells (in green) activate JNK signalling. This induces the production of ROS (in red) and the attraction of hemocytes (in blue) into the transformed areas (green). Upon interaction with the tumour, hemocytes become activated, releasing the TNF ligand Eiger (in magenta). Eiger goes on to stimulate further JNK activity, creating a positive feedback loop that promotes tumour growth and inflammation.

Along these lines,* Drosophila *investigations have correlated the expansion of genetically induced tumours with the recruitment of tumour associated hemocytes (TAHs) and their production of Eiger (TNF-*α* in flies) [191, 192]. Although the pioneering works were not able to identify the molecular mechanisms essential for TAH recruitment, recent data has shown the requirement of Dronc during this process [193]. In the induced-cancer cells, the upregulation of JNK signalling prompts non-apoptotic caspase activity, which ultimately stimulates reactive oxidative species (ROS) production [193]. ROS production is a potent hemocyte recruitment factor [194, 195] that attracts these immune cells towards areas with transformed cells [193]. Hemocytes can then interact with the tumour cells and produce Eiger, which further stimulates JNK activity in cancer cells [191]. All of these events close a positive feedback loop that promotes tumour growth [193]. This cancer model beautifully illustrates the interplay between caspases and the immune system (Figure 4), while confirming the power of this model organism for uncovering fundamental aspects of cancer [17].

Interestingly, inflammation and signals released from inflammatory cells, such as ROS, are able to touch upon another enabling characteristic of tumorigenesis: genome instability and mutations [1]. ROS and other chemicals released from inflammatory cells are actively mutagenic, quickening the genetic evolution of cancer cells towards malignancy through DNA damage [164]. Similarly, DNA damage caused by sublethal levels of caspase activity has been shown to promote genome instability and carcinogenesis, through the activation of endonucleases such as endonuclease G (EndoG) and caspase-activated DNase (CAD) [196–198]. Paradoxically, CAD-induced DNA damage can also regulate the differentiation of myoblasts in physiological conditions [199]. Although there is no direct evidence connecting the activity of DNases with tumorigenesis in* Drosophila* models, the evolutionary conservation of these proteins [200] suggests that* Drosophila* could be used to investigate the role of caspase-induced DNA damage in carcinogenic processes.

Inflammatory caspases in mammals have also been demonstrated to partake in inducing cell proliferation in normal and cancer cells. The literature is vast on this subject and outside of the scope of this review [113, 163–165]; however, here we provide a few selected examples. Colonic epithelial cells show increased proliferation and reduced apoptosis when deficient for caspase-1 [201]. Caspase-11 has been also implicated in promoting intestinal epithelial cell proliferation through the inflammasome-meditated cleavage of the proinflammatory cytokine IL-18 [202]. Importantly, defective signalling from the inflammasome has been shown to contribute to colitis, but also colorectal tumorigenesis, through loss of intestinal barriers and aberrant proliferation [203]. These studies collectively described the complex intersection between caspase signalling and the immune response, while highlighting its decisive role in the appearance and clonal expansion of cancerous cells.

## 5. Therapeutic Potential of Caspase Modulation and Drosophila as a Vehicle for Drug Discovery

The enzymatic nature of caspases and their ability to regulate the process of apoptosis has attracted the interest of pharmaceutical companies to discover compounds with caspase-modulating activity. Indeed, there are a substantial number of apoptotic-regulatory compounds in preclinical or phase trials for treating specific diseases [34, 204, 205]. However, several factors have traditionally hampered the transition of such molecules from the bench to bedside. From the therapeutic perspective, the desired adjustment to caspase-kinetics appears dependent upon the underlying pathology and is not always easy to attain both* in vitro* and* in vivo*. Whereas studies by Akpan and collaborators demonstrated that inhibition of caspase-9 was neuroprotective after stroke [206], other studies have conversely demonstrated the efficacy of promoting a pro-apoptotic response during cancer therapy [204] to facilitate the elimination of cell death resistant cancerous cells. Several concerning side effects have also been detected upon treatment with pro-apoptotic agents. Recent studies have reported an increased risk of bone metastasis and osteoporosis linked to these therapies, as well as undesirable side effects due to low compound specificity [207, 208]. Finally, caspase-modulating molecules can impact the inflammatory response with highly diverse consequences occurring depending on the cellular context [208]. Altogether, the evidence highlights the therapeutic potential of caspase-modulating molecules, while stressing the need to anticipate side effects through research in complex cellular models.

**Figure 5:**
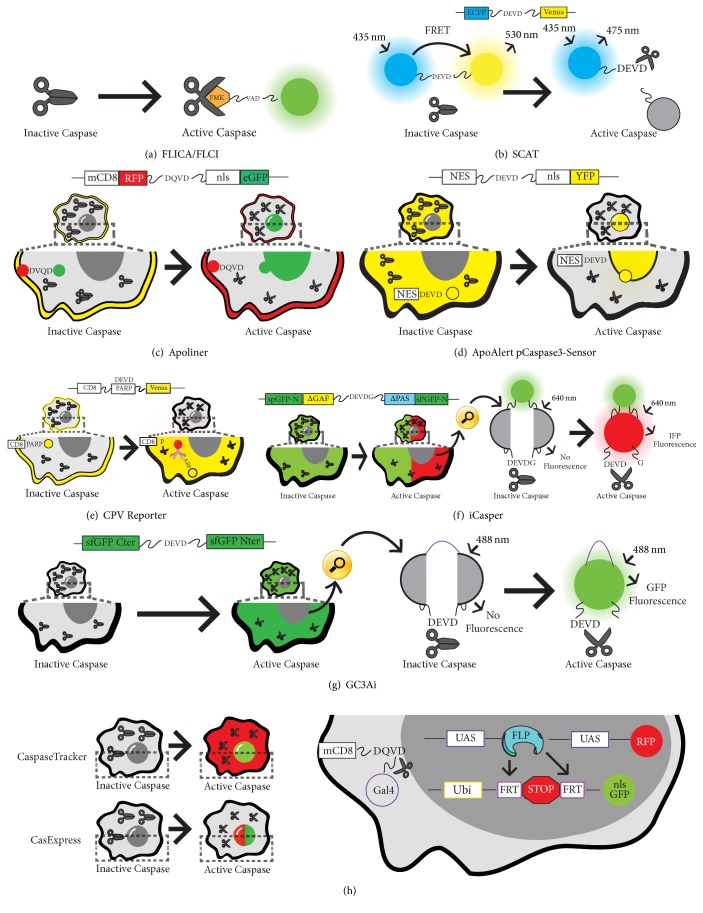
Rational design and activity of different caspase sensors. (a) Caspase visualization based on artificial fluorochrome labelled inhibitors (FLICA and FLCI). (b) Schematic diagram that shows the SCAT FRET. When caspases are inactive the Enhanced Cyan Fluorescent Protein (ECFP) through Fluorescence Resonance Energy Transfer (FRET) allows the fluorescence of a Venus fluorescent protein at 530 nm (b). Upon caspase activation cleavage of the tethering sequence occurs, resulting in FRET signal loss and fluorescence emission at 475 nm (b). (c) Schematic diagram of Apoliner. This reporter is tethered to cellular membranes through a consensus CD8 membrane anchor domain. Upon caspase activation a DQVD sequence is cleaved (c), releasing a GFP moiety that is translocated into the nucleus; however, the red fluorescent signal RFP is retained at the cellular membrane upon caspase activation (c). (d) ApoAlert pCaspase3-Sensor. The Yellow Fluorescent signal (YFP) is initially retained in the cytoplasm, but a nuclear localization signal (NLS) allows the translocation into the nucleus upon caspase activation (d). (e) CPV reporter. Caspase reporter containing a Venus fluorescent protein tethered to the intercellular membrane through a consensus CD8 sequence. Upon caspase activation the caspase-recognition linker contained in the PARP protein enables the diffusion of Venus-FP into the cytoplasm. The cleaved PARP conjugated to the Venus fluorescent protein can be recognized by an Anti-parp antibody (e). (f) Schematic diagram of iCasper reporter. This reporter consists of two segments of a split GFP protein tethered by a linking region, in addition to a separated infrared fluorescent protein containing the caspase cleavage sequence: DEVD. The presence of the DEVDG linker separates the infrared fluorescent protein (IFP) inhibiting its light emission. Caspase activation results in cleavage of the consensus sequence, allowing IFP fluorescence following excitation of 640 nm. (g) Schematic representation of iGC3 reporter. This reporter consists of two segments of a green fluorescent protein (GFP) tethered by a caspase cleavage recognition sequence, DEVD. Upon caspase activation, the DEVD sequence is cleaved allowing the interaction of both GFP fragments and subsequent fluorescent emission (f). (h) CaspaseTracker and CasExpress. A CD8 sequence tethers a DQVD caspase cleavage sequence and a Gal4 transcription factor to the intracellular membranes. Caspase activation results in cleavage of the sequence and Gal4 transport into the nucleus (h). Gal4 then can activate cell markers with variable protein perdurance upon binding to UAS sequences (e.g., RFP cytoplasmic signal) (h). Additionally, it produces a flippase recombinase that mediates the excision of a stop cassette flanked by FRT sites. Upon excision a permanent marker (nuclear GFP) is expressed under the regulation of a constitutive promoter (Ubiquitin), resulting in a permeant labelling of caspase-activating cells (h). CasExpress has the same rational design as CaspaseTracker; however the authors used a nuclear RFP for showing short-term activation of caspases, instead of a cytoplasmic marker. In all panels black scissors can represent either active or inactive caspases (open or closed, resp.).

*Drosophila melanogaster* has recently emerged as an excellent model for drug discovery and the evaluation of compound pharmacodynamics [209–212]. For example, methotrexate, gemcitabine, and topotecan are all FDA approved compounds originally validated and/or developed in* Drosophila *[213–215]. Until recently, screening for caspase-modulating chemotherapeutics in* Drosophila *was problematic, owing to the absence of* in vivo* tools able to monitor caspase activity using a high-throughput approach. Historically, measurement of* in vivo* caspase activation was achieved through the cellular application of fluorescently tagged, small non-reversible binders of activated caspases (Figure 5(a)) [216, 217]. Despite the short half-life of these compounds, concerns were raised regarding the biological significance of these molecules in physiological conditions. Luciferase reporters were then developed; however, they suffered from similar criticisms [218]. One of the pioneering breakthroughs in the* in vivo *monitoring of caspase activation in* Drosophila* came with the publication of the SCAT reporter [219, 220]. The SCAT sensor consists of two fluorophores suitable for FRET microscopy linked via a short caspase cleavage site specifically recognized by effector caspases (ECFP-DEVD-Venus). The expression of the sensor in Hela cells and* Drosophila* tissues reliably detected caspase activation upon caspase cleavage in lethal and non-lethal scenarios (Figure 5(b)) [219, 220]. Since then, the toolkit in flies of caspase sensors has significantly been expanded. Although all subsequent sensors have maintained a core caspase-recognition site for effector caspases, multiple combinations of flanking fluorophores have conferred upon them different capabilities (Figures 5(b)–5(h)). One of the sensors described after SCAT included two fluorescent proteins that change their subcellular localization upon caspase cleavage (Apoliner [CD8-RFP-DQVD-nlsGFP]) (Figure 5(c)) [221]. ApoAlert pCaspase3-Sensor (NES-DEVD-YFP-NLS) was another reporter based upon changes in the subcellular localization of fluorescence (Figure 5(d)) [222]. Alternatively, other sensors exploited the immunoreactivity of specific epitopes upon caspase-mediated excision for detecting caspase activation (CD8-PARP-Venus) (Figure 5(e)) [223]. More advanced and recent methods have used split fluorescent proteins that only fluoresce upon caspase-mediated excision of the short linker joining the two subunits of the fluorophore (Figures 5(f) and 5(g)) [224, 225]. Highly sensitive sensors like these are able to potentially detect caspase activation with subcellular resolution in* Drosophila* tissues (Figure 5(g)) [225]. Finally, new sensors have been published with ability to provide a temporal perspective of caspase activation. The rational design of these sensors includes a transcriptional activator (Gal4) that is released from the cellular membranes upon caspase-mediated cleavage of a short caspase-recognition motif. Once in the nucleus, Gal4 can drive the expression of transient or permanent cellular markers under the regulation of Upstream-Activating-Sequences (UAS) (Figure 5(h)) [226, 227]. These sensors have proven extremely useful for detecting the presence of caspase-activating cells that do not enter the apoptotic program, while enabling their genetic manipulation. Although only some of these sensors are truly suitable for high-throughput drug screens, they promise to bring new opportunities in the coming years for uncovering the effects of caspase-modulating molecules in complex* Drosophila* settings. Furthermore, they could potentially help to anticipate obvious pharmacological complications such as tissue toxicity, compound clearance properties, and tissue targeted delivery.

## 6. Conclusion

**Figure 6:**
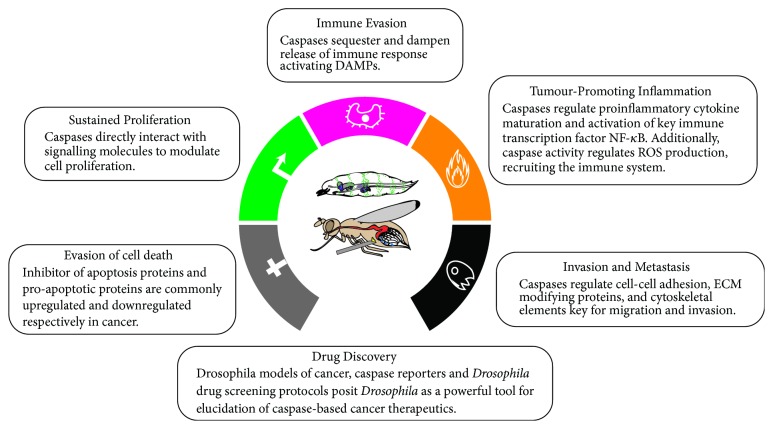
Schematic diagram summarizing the implication of caspases in many hallmarks of cancer. Modified from Hanahan and Weinberg, 2011.

In this review, we have highlighted how the roles of caspases extend far beyond their canonical functions during apoptosis, in either normal or tumorigenic scenarios. Along this line, we have discussed the latest evidence indicating the critical roles of caspases in the regulation of fundamental biological processes and how caspase malfunction contributes to almost all aspects of tumorigenesis (summarized in Figure 6). We hope to have illustrated that although there has been much progress, the molecular mechanisms behind these newly identified caspase roles are still largely unclear. More research should be undertaken in order to fully understand caspase biology and its connection to tumour development. Finally, we have shown that many of the findings discussed in the manuscript have emerged from research conducted in the simple but genetically powerful model organism* Drosophila melanogaster*. Indeed, given the previously stated advantages of research in flies, we consider this model organism uniquely positioned to studying the intersection between caspases and cancer, as well as uncovering novel compounds aimed at modulating caspase activity from a therapeutic perspective.
